# 0714. Interaction between adipokines and the metabolic stress response: angpt l2 cxcl5 and visfatin in patients undergoing cardiac surgery

**DOI:** 10.1186/2197-425X-2-S1-P46

**Published:** 2014-09-26

**Authors:** T Meyer, E Nitschmann, U Schurr, A Boltres, C Bläsi, L Wykes, H Pargger, A Kopp Lugli

**Affiliations:** Department of Anesthesia & Surgical Intensive Care, University Hospital Basel, Basel, Switzerland; School of Dietetics and Human Nutrition, McGill University, Montreal, Canada; Department of Cardiac Surgery, University Hospital Basel, Basel, Switzerland

## Introduction

Adipose tissue plays an intriguing role in the endocrine system by producing adipokines [1]. In patients with obesity, metabolic syndrome, and type 2 diabetes (DM2), insulin resistance is influenced by adipokines [1].

From a metabolic point of view, the stress response caused by surgical injury imitates the diabetic state. Stereotypical endocrine-metabolic and inflammatory reactions result in catabolism and pronounced insulin resistance, which is related to outcome [2].

## Objectives

Three adipokines including angiopoietin-like protein 2 (ANGPTL2), CXC-chemokine ligand 5 (CXCL5), and Visfatin that are known to interact with insulin resistance [1] were evaluated in patients undergoing cardiac surgery.

## Methods

Sixty-six patients scheduled for elective aortocoronary bypass surgery and/or valve repair were consecutively enrolled receiving standardized perioperative care. Serum adipokine concentrations were assessed before anesthesia induction as baseline values, upon arrival on the intensive care unit, on the first (POD1) and third (POD3) day postoperatively.

## Results

Patients' baseline characteristics are shown in table 1. ANGPTL2 increased from baseline until POD1 and remained elevated on POD3 (table 2). In contrast, CXCL5 levels decreased during surgery and returned to baseline by POD3. Visfatin levels increased during surgery, showed a decrease by POD1 only to increase again by POD3. DM2 patients appear to exhibit a marked Visfatin increase during surgery, as well as a pronounced increase in CXCL5 from POD1 to POD3.

**Table 1:** Table 1

Variable	All Patients (66)	Non-Diabetics (46)	Diabetics (20)
Sex (M/F)	45/21	33/13	12/8
Age (yrs)	65.5 ± 13	64 ± 14	68.7 ± 9.8
ASA (2/3/4)	13/46/7	9/35/2	4/11/5
BMI (kg/m2)	27.5 ± 4.4	26.4 ± 4.2	30.1 ± 3.9
HbA1c (%)	6.3 ± 0.9	5.9 ± 0.4	7.3 ± 1.1
EuroSCORE	4.8 ± 4.6	4.0 ± 2.6	6.5 ± 7.1
SAPS II Score	28.3 ± 8.3	27.7 ± 8.7	29.9 ± 7.1
Type of surgery AKB/valve/multiple	23/22/21	16/17/14	7/5/7
Duration of surgery (min)	212.5 ± 47.2	206.2 ± 46.2	226.8 ± 47.5

**Table 2:** Table 2

Variable		Preop	Arrival ICU	POD 1	POD 3
ANGPTL2 (ng/ml)	All	66 ± 36	86 ± 30	96 ± 35	90 ± 35
	Non-DM2	71 ± 38	85 ± 32	97 ± 36	90 ± 38
	DM2	56 ± 29	87 ± 26	93 ± 31	90 ± 30
CXCL5 (pg/ml)	All	1022 ± 503	662 ± 357	742 ± 456	936 ± 595
	Non-DM2	1009 ± 479	620 ± 331	695 ± 380	890 ± 532
	DM2	1051 ± 563	756 ± 402	855 ± 598	1043 ± 723
Visfatin (ng/ml)	All	4.9 ± 2.5	13.2 ± 7.1	7.7 ± 3.4	12.6 ± 5.8
	Non-DM2	4.6 ± 2.4	12.5 ± 7.2	7.6 ± 3.6	12.6 ± 6.2
	DM2	5.3 ± 3.2	14.6 ± 6.9	7.8 ± 2.9	12.5 ± 4.9

## Conclusions

This exploratory study assessed for the first time the perioperative levels of ANGPTL2, CXCL5, and Visfatin. Further studies are warranted to correlate adipokine concentrations with insulin resistance and surgical outcomes and to investigate the potential role of adipokines as biomarkers or therapeutic targets.
